# Supplementary material for: ProteinShader: illustrative rendering of macromolecules
Source: BMC Struct Biol. 2009 Mar 30;9:19. doi: 10.1186/1472-6807-9-19 (PMC2672931; doi:10.1186/1472-6807-9-19)
Supplement: Additional file 1 — ProteinShader program without source code. This compressed file contains the complete ProteinShader program including associated libraries, but no source code. A README.txt file gives an overview of the ProteinShader distribution, and the index.html file in the help subdirectory has directions on getting started with the program as well as a set of tutorials. [file 1472-6807-9-19-S1.zip › ProteinShader-beta-0_9_4-binary/help/api/org/proteinshader/structure/enums/class-use/HelixShapeEnum.html]

Uses of Class org.proteinshader.structure.enums.HelixShapeEnum (ProteinShader API)


|  |  |  |
| --- | --- | --- |
| **Overview** \| **Package** \| **Class** \| **Use** \| **Tree** \| **Deprecated** \| **Index** \| **Help** | |  |
| PREV   NEXT | **FRAMES**    **NO FRAMES**     **All Classes** |  |


---


## **Uses of Class org.proteinshader.structure.enums.HelixShapeEnum**

| Packages that use HelixShapeEnum | |
| --- | --- |
| **org.proteinshader.structure** | Holds the classes that store information from a Protein Data Bank file: Structure, Model, Chain, AminoAcid, Heterogen, Water, Atom, Bond, Helix, BetaStrand, Loop, *etc*. |
| **org.proteinshader.structure.enums** | Holds the enums needed by org.proteinshader.structure and its subpackages. |

Uses of HelixShapeEnum in org.proteinshader.structure

| Fields in org.proteinshader.structure declared as HelixShapeEnum | |
| --- | --- |
| `static HelixShapeEnum` | `Helix.DEFAULT_SHAPE`             The default Helix shape is ALPHA\_RIBBON. |

| Methods in org.proteinshader.structure that return HelixShapeEnum | |
| --- | --- |
| `HelixShapeEnum` | `Helix.getShape()`             Returns the shape that should be used to represent the Helix. |

| Methods in org.proteinshader.structure with parameters of type HelixShapeEnum | |
| --- | --- |
| `void` | `Helix.setShape(HelixShapeEnum shape)`             Sets the shape that should be used to represent the Helix. |

Constructors in org.proteinshader.structure with parameters of type HelixShapeEnum

`Helix(String helixID,
String serialNo,
String startResidueID,
String endResidueID,
Chain chain,
HelixEnum type,
HelixShapeEnum shape)`
  
          Constructs a Helix.

Uses of HelixShapeEnum in org.proteinshader.structure.enums

| Methods in org.proteinshader.structure.enums that return HelixShapeEnum | |
| --- | --- |
| `static HelixShapeEnum` | `HelixShapeEnum.valueOf(String name)`             Returns the enum constant of this type with the specified name. |
| `static HelixShapeEnum[]` | `HelixShapeEnum.values()`             Returns an array containing the constants of this enum type, in the order they're declared. |

---


|  |  |  |
| --- | --- | --- |
| **Overview** \| **Package** \| **Class** \| **Use** \| **Tree** \| **Deprecated** \| **Index** \| **Help** | |  |
| PREV   NEXT | **FRAMES**    **NO FRAMES**     **All Classes** |  |


---

# *Copyright © 2007-2008*
